# An Oxford Nanopore Technologies–Based Sequencing Assay for Molecular Diagnosis of Phenylketonuria and Variant Frequencies in a Turkish Cohort

**DOI:** 10.1155/ijog/5552662

**Published:** 2025-04-25

**Authors:** Gülten Tuncel, Mehmet Cihan Balcı, Gökçe Akan, Hasan Hüseyin Kazan, Özge Özgen, Ahmet Çağlar Özketen, Meryem Karaca, Asuman Gedikbaşı, Fatmahan Atalar, Gülden Fatma Gökçay

**Affiliations:** ^1^DESAM Research Institute, Near East University, Mersin, Türkiye; ^2^Division of Paediatric Nutrition and Metabolism, Istanbul Medical Faculty, Istanbul University, Istanbul, Türkiye; ^3^Department of Medical Biology, Gulhane Faculty of Medicine, University of Health Sciences, Ankara, Türkiye; ^4^Rare Diseases Research Laboratory, Istanbul Medical Faculty, Istanbul University, Istanbul, Türkiye; ^5^Department of Paediatric Basic Sciences, Institute of Child Health, Istanbul Medical Faculty, Istanbul University, Istanbul, Türkiye; ^6^Department of Rare Diseases, Child Health Institute, Istanbul University, Istanbul, Türkiye

**Keywords:** molecular diagnostic techniques, nanopore sequencing, neonatal screening, phenylalanine hydroxylase, phenylketonurias

## Abstract

**Background:** Phenylketonuria (PKU) is an autosomal recessive metabolic disorder caused by mutations in the *PAH* gene, resulting in deficient phenylalanine hydroxylase (PAH) enzyme activity and neurotoxic phenylalanine accumulation. Untreated PKU results in progressive neurodegeneration and severe intellectual disability. Neonatal screening has evolved from the Guthrie test to advanced techniques like HPLC, tandem mass spectrometry, and next-generation sequencing (NGS) for molecular confirmation. This study aimed to develop a rapid, scalable *PAH* genetic assay using Oxford Nanopore Technologies (ONTs) to enhance neonatal screening in high-prevalence regions like Türkiye, through accelerated, cost-effective genetic diagnostics.

**Methods:** An in-house panel was designed, implemented, and benchmarked against results obtained from the Illumina sequencing platform. A cohort of 40 PKU patients, previously diagnosed using Illumina platform, was selected for this study. Gene-specific primers were strategically designed to amplify exonic regions, untranslated segments, and exon–intron junctions of the *PAH* gene. Sequencing libraries were then prepared and processed using the MinION Mk1c instrument, with subsequent data analysis conducted through the Guppy software and complementary bioinformatics tools.

**Results:** The findings showed complete agreement between the ONT and Illumina platforms, corroborating the high fidelity and reliability of the ONT-based assay. All pathogenic variants previously identified through Illumina sequencing were accurately detected, albeit with varying observed allele frequencies. Notably, the most prevalent variants identified in the patient cohort were NC_000012.12(NM_000277.3):c.1066-11G > A with a frequency of 37.5% and NC_000012.12(NM_000277.3):c.782G > A, at 15%.

**Conclusion:** The ONT-based single-gene testing for PKU demonstrated complete concordance with Illumina sequencing, validating its accuracy and reliability. This method effectively detects pathogenic variants and offers a faster, cost-effective solution for neonatal screening, particularly beneficial in high-prevalence regions like Türkiye.

## 1. Introduction

Phenylketonuria (PKU), also known as phenylalanine hydroxylase (PAH) deficiency (OMIM #261600), is an autosomal recessive disorder characterized by mutations in the *PAH* gene. The PAH enzyme (EC 1.14.16.1) encoded by *PAH* gene normally catalyzes the hydroxylation of Phe to tyrosine, a crucial step in the catabolism of dietary proteins and other sources of Phe. Under physiological conditions, PAH enzyme plays an important role in eliminating approximately 75% of the Phe obtained from dietary sources and catabolism of the proteins. The expression of PAH is high in the liver where excess amount of Phe is removed. However, in individuals with PKU, the deficient PAH enzyme fails to adequately metabolize Phe, resulting in its accumulation in the bloodstream and leading to its potentially toxic metabolites. This pathophysiological condition, known as hyperphenylalaninemia (HPA), is the most severe form in PKU when pretreatment serum Phe levels are more than > 1200 *μ*M. In PKU, excessive Phe and its toxic metabolites interfere with neurotransmission in the brain, causing neurological complications such as mental retardation and involuntary movements [1–3]. Symptoms of PKU typically arise within the first few months of life, though they can be subtle initially. If left untreated, elevated Phe levels can lead to severe intellectual disability, developmental delays, behavioral problems, and neurological complications such as seizures and microcephaly. Additionally, untreated individuals with PKU may exhibit lighter skin and hair compared to their family members due to decreased melanin synthesis [4]. The spectrum of symptoms can vary widely, with some individuals experiencing milder forms of the disorder that still require treatment to prevent cognitive impairment [3]. Once diagnosed, the treatment of PKU primarily requires a strict, lifelong dietary regimen designed to restrict Phe intake. Recent advancements in PKU treatment include sapropterin dihydrochloride (BH4) supplements that boosts enzyme activity in some patients, and other options such as LNAA supplements, which help lower Phe levels. These treatments represent avenues for enhancing the management of PKU beyond dietary restrictions alone [5–7].

The prevalence of PKU varies globally, with incidence rates typically around 1 in 10,000–15,000 births in the United States and Europe. However, in certain regions such as Türkiye and Ireland, higher prevalence rates have been observed, attributed to genetic factors and increased consanguinity [8–10]. PKU was the first disease to be identified by the neonatal screening programs. These population-based screening initiatives have significantly improved PKU detection and management, enabling early intervention and significantly reducing the disease burden [11].

Over the years, diagnostic methods for PKU have significantly evolved, from the simple ferric chloride urine test to the Guthrie test enabling the neonatal screening to become a pivotal public health measure in several countries. Screening typically involves the measurement of Phe levels in dried blood spots collected shortly after birth, followed by confirmatory testing using quantitative amino acid analysis or Sanger sequencing [12]. Initially the Guthrie test, a semiquantitative method with limited sensitivity was employed. This was subsequently replaced by more sensitive and precise techniques such as quantitative fluorometric methods, and consecutively by tandem mass spectrometry (MS) technology, which enhanced the accuracy and efficiency of screening programs [13].

In parallel, over the last decade, next-generation sequencing (NGS) systems have gained prominence in genetic diagnosis. Nevertheless, in practice, the diagnosis of PKU still primarily relies on blood Phe levels. Genetic analyses are conducted to assess tetrahydrobioterin (BH4) responsiveness, predict the clinical form of the disease, and differentiate BH4 metabolism disorders. NGS allows comprehensive analysis of the *PAH* gene, enabling the identification of known and novel mutations with high accuracy and speed which was not possible with MS/MS. Unlike traditional methods, this advancement has facilitated earlier detection, precise characterization of genetic variants, and tailored management strategies for individuals affected by PKU [14, 15]. However, despite their widespread adoption in neonatal screening, NGS can encounter challenges, especially in the time-consuming nature of the library preparation [16]. Third-generation NGS systems, exemplified by Oxford Nanopore Technologies (ONTs) present a promising solution to the limitations associated with earlier, second-generation NGS platforms [17]. ONT systems have the potential to reduce both costs and labor requirements, addressing efficiency concerns and enhancing the feasibility of genetic screening and diagnostic applications for conditions like PKU.

In the present study, our objective was to develop an ONT-compatible approach for single-gene testing in the molecular diagnosis of PKU. We employed an in-house panel specifically designed for screening clinical samples and evaluated its performance by comparing it with the Illumina sequencing platform. This approach aims to explore the potential of ONT systems in human genetics applications, particularly for diagnosing inherited metabolic disorders, thereby representing an innovative step towards advancing neonatal screening.

## 2. Methods

### 2.1. Study Design and Population

This study was designed as a method development and validation study. Then, 40 patients were recruited from the Istanbul University, Istanbul Medical Faculty, Department of Pediatrics, Division of Nutrition and Metabolism between 2021 and 2024 years for this study, which was structured as a test validation approach. The present study was approved by Istanbul Medical Faculty Ethics Committee under the number 23.11.2022-1397546. Verbal and written consents were obtained from each patient or their guardian. All patients had previously been diagnosed with PKU based on quantitative Phe results determined by high-performance liquid chromatography (HPLC), and molecular diagnoses had been conducted using an Illumina-based targeted panel. Prior to collecting peripheral blood samples for molecular studies, demographic information listed in Table 1 was obtained. Total genomic DNA was isolated from the participants blood using QIAmp DNA Blood Mini kit (Qiagen, Hilden, Germany) following the manufacturer's protocol.

### 2.2. Primer Design and Optimization

**Figure 1:**
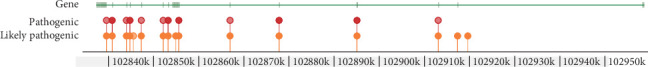
Genomic distribution of the pathogenic and likely pathogenic variants on the *PAH* gene. Adapted from the NCBI ClinVar database (https://www.ncbi.nlm.nih.gov/clinvar/).

The primers designed to amplify the exons, untranslated regions (UTRs), and exon–intron boundaries of the *PAH* gene (NM_000277.3) in which pathogenic or likely pathogenic variants have already been reported (Figure 1) were generated using the NCBI Primer-BLAST tool [18]. A total of nine primer pairs were designed to produce amplicons of approximately 1500 to 5000 base pairs in size. Prior to pooling for patient sample sequencing, the designed primers underwent analysis using *in silico* PCR (Silica; Gear Genomics; https://www.gear-genomics.com/silica/). This computational approach enabled virtual PCR amplification of the target regions to predict and validate primer specificity and efficiency before experimental implementation.

For primer optimization, human reference DNA (Agilent Technologies, United States) was used as a positive control, while a negative control containing all reaction components except template DNA was included to assess potential contamination and ensure the specificity of the amplification. Each primer was used at a final concentration of 0.1 *μ*M for amplification of target regions, using 2X Hi-Fi enzyme master mix (MobiomX, Türkiye) and 35 ng of DNA. The optimized thermal cycling profile included an initial denaturation at 95°C for 3 min, followed by 40 cycles of denaturation at 95°C for 15 s, annealing at 66°C for 45 s and extension at 72°C for 4 min; and a final extension step at 72°C for 10 min concluded the thermal cycling process. PCR products were visualized by gel electrophoresis on a 1% agarose gel, confirming the presence of the DNA bands of the expected size, while the negative control exhibited no amplification, validating the reaction's specificity. Subsequently, the primers were pooled into two tubes based on rational considerations. Following primer pooling, PCR was conducted using human reference DNA and negative controls. A representative image of the gel electrophoresis results is provided in Figure S1. The resulting amplicons were then subjected to sequencing. After sequencing, coverage and read depths were checked and primer pairs were exchanged between the pools to address any amplification issues, ensuring uniform coverage of the *PAH* gene. Patient samples were then screened using the optimized primer pools and sequencing protocols described above.

### 2.3. Library Preparation and ONT Sequencing

Sequencing libraries were prepared for this study using the following materials and equipment: SQK-LSK109, and EXP-NBD196 kits, FLO-MIN106D flow cell, and Mk1C device from ONTs (Oxford, United Kingdom). PCR amplicons were initially purified using AMPure XP beads (Beckman Coulter Life Sciences, United States), and subsequently, they underwent end-repair using the NEBNext Ultra II End-Repair/dA-tailing Module (New England Biolabs, United States), followed by barcoding. After another purification step to ensure high quality, the products were adapter-ligated using Adapter Mix II. After a final purification step and determination of the concentrations using Qubit 4 Fluorometer (Thermo Scientific, United States), 400 ng of the libraries was loaded onto the MinION Mk1c device and sequenced for a duration of 48 h.

### 2.4. Data Analysis

In the ONT platform, Guppy software was used for the basecalling and demultiplexing of sequencing data. Raw Fastq files underwent processing using Massive Analyser (Massive Bioinformatics, Türkiye), which included quality assessment using FastQC (http://www.bioinformatics.babraham.ac.uk/projects/fastqc/) embedded within the Massive Analyser program. To align reads to the human reference DNA sequence, an in-house bed file was utilized to correct alignment errors with MiniMap2 [19]. Medaka (https://github.com/nanoporetech/medaka) was then employed to correct the alignment errors. Variant calling was performed using Clair3-Trio [20], and subsequent variant annotation was conducted with ANNOVAR [21]. The identified variants were filtered using VarAFT software [22] and prioritized by Franklin by Genoox (https://franklin.genoox.com) according to the American College of Medical Genetics (ACMG) criteria [23]. Genotype–phenotype correlations were established using entries from the BioPKU database [24] and biochemical classification of PKU (classic, mild, or undefined), as previously described by Nalin et al. [25].

## 3. Results

### 3.1. Patients and the Demographic Information

The present study enrolled 40 patients diagnosed with PKU, whose molecular diagnosis had been previously conducted using Illumina-based targeted sequencing. Clinical diagnosis of PKU was done according to patient Phe levels, as classic PKU (Phe > 1200*  μ*mol/L) and mild PKU (Phe 600–1200 *μ*mol/L). Accordingly, the patient group included 31 classic PKU (77.5%) and 9 mild PKU (22.5%) patients. These patients were currently undergoing dietary treatment. Among these patients, 18 (45%) were female and 22 (55%) were male. Of the 40 patients, clinical diagnosis of 30 were in concordance with the BioPKU data. Their ages ranged between 1 and 37 years old, reflecting the diverse age distribution of follow-up patients. According to the questionnaire, which had missing data for four patients, it was found out that the parents of 17 patients (47.2%) were consanguineous (Table 1).

### 3.2. Assay Optimization and Sequencing Quality Assessment

**Figure 2:**
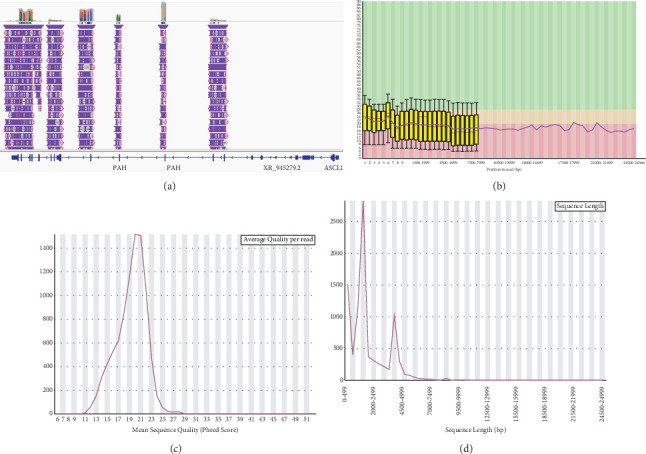
Quality assessment of the sequencing data. (a) Coverage visualization using the IGV. (b) Quality scores across all bases. (c) Distribution of quality scores across sequences. (d) Distribution of sequence lengths across sequences.

The ONT-based *PAH* screening assay was developed to amplify the exons, UTRs, and exon–intron boundaries using 9 primer pairs. After primary and singularly evaluation of the primer pairs by human reference DNA, the primers were pooled into two tubes. Subsequently, these pooled primers underwent further validation using human reference DNA, involving gel electrophoresis and ONT-sequencing. The sequencing quality was assessed using FastQC, and read visualization and analysis were performed using the Integrative Genomics Viewer (IGV) tool [26]. According to the findings (Figure 2), all targeted genomic regions; exons, UTRs, and exon–intron boundaries, were effectively covered with varying read depths ranging from 50 to 2000x. Moreover, the read quality was observed to decrease slightly for sequences in the range of approximately 1500 to 5000 bps. However, this characteristic was deemed suitable and advantageous for subsequent screening of the patient samples.

### 3.3. Variant Evaluation

Following assay optimization using human reference DNA, the developed system was applied to screen *PAH* gene in PKU patients previously diagnosed using an Illumina-based sequencing panel. The results pointed out that the newly developed assay effectively detected all pathogenic variants already identified by the Illumina sequencing (Table 1). However, the allele frequency necessary to determine homozygosity was relatively low, specifically greater than or equal to 0.58.

Among the patients, the intronic variant, NC_000012.12 (NM_000277.3):c.1066-11G > A exhibited a high frequency, detected in 15 patients (15/40; 37.5%). Among these, six patients were homozygous, eight patients were compound heterozygous, and one patient was singularly heterozygous for this mutation. The second most frequently detected variant was NC_000012.12 (NM_000277.3):c.782G > A (6/40; 15%) found in six patients, with two patients demonstrating a homozygous pattern and four patients being heterozygous. Importantly, 17 patients (42.5%) had pathogenic/likely pathogenic variants in a compound heterozygous configuration, while three patients (7.5%) possessed a single heterozygous variant insufficient to explain the genetic basis of the disease.

## 4. Discussion

In the present study, we designed a novel in house amplification-based *PAH*-screening panel adaptable to one of the long-read sequencing platforms, ONT. Pooling nine primer pairs covering all exonic, splicing, and mutation-reported deep intronic regions and UTRs of the *PAH* gene into two tubes, we successfully optimized the system using reference human DNA. By optimized system, we screened 40 PKU patients who were already molecularly diagnosed by Illumina platform. Our results pointed out a 100% correlation with clinical reports of the patients, emphasizing the success rate of the in-house ONT system in clinical genetics in terms of molecular diagnosis of PKU.

Early and accurate diagnosis of PKU is crucial given its treatable nature. Biochemical and analytical methods are typically applied for the neonatal screening of PKU, making it a standard inclusion in neonatal screening programs across many countries [27]. However, conventional screening methods may be misleading, particularly when the levels of Phe are elevated due to other inherited metabolic disorders involving deficiencies in tetrahydrobiopterin metabolism [3]. MS/MS is the method of choice for neonatal screening, yet the high ratio of false positive results significantly restricts its applicability [28]. Thus, molecular diagnosis plays a pivotal role in ensuring accurate differential diagnosis of PKU. Thus, sequencing *PAH* gene using an NGS platform offers distinct advantages in terms of rapid, cost-effective, and accurate screening for PKU. By biochemical or analytical methods, PKU can be diagnosed; nonetheless, the severity and treatment of the disease could be heterogenous depending on the affected member of the tetrahydrobiopterin metabolism. Thus, analytical methods such as MS/MS could primarily be used for the diagnosis of PKU; nevertheless, molecular methods including NGS as a second-tier testing are required for differential diagnosis and accordingly proper treatment approaches [29]. Among the NGS platforms, so-called second-generation systems are predominantly used systems for the molecular diagnosis of PKU [30, 31]. However, owing to having a feature to read the target gene regions as small fragments, these systems may fail to detect large deletions and/or duplications reported with high frequencies in the *PAH* gene [32]. Moreover, the establishment of the NGS sequencing facilities and the associated per-sample cost can be relatively high, posing challenges for equal accessibility in local regions, and potentially limiting compliance with regulatory requirements for the neonatal screening programs [33, 34]. ONT platform offers a practical and effective alternative in terms of time and cost burden in diagnostic screening [35]. Hence, we study long-read sequencing capacity of ONT to improve differential diagnosis of PKU for adoption in second-tier genetic analysis.

According to the results obtained in the study, the amplicon sizes aligned with the expectations set by the primer designing strategy, typically ranging from 1500 to 5000 bp. This alignment was confirmed through both the ONT sequencing and the gel electrophoresis. All targeted regions, including exons, UTRs, and exon–intron boundaries of the *PAH* gene, were successfully covered by our assay. However, the quality scores of the reads obtained from ONT sequencing were lower compared to the data generated by Illumina platforms (http://www.bioinformatics.babraham.ac.uk), which is a known characteristic of ONT sequencing. Despite this, the read qualities were relatively high when compared to the studies reported in existing literature [36]. Hence, we regarded to further validate the developed system by the patient samples.

The validation of the system using patient samples, who were previously diagnosed using an Illumina-based assay, focused on detecting the pathogenic/likely pathogenic variants. According to the results, the read depths across the target regions varied, and adjustments in primer design and/or input concentrations did not resolve this variability, potentially attributed to the challenges associated with long PCR applications [37]. Nevertheless, the minimum read depth exceeded 50x which was deemed adequate for the molecular analyses [38]. Importantly, the variants that were detected by the developed ONT-based systems showed a 100% correlation with those identified by the Illumina-based assay. When comparing variant allele frequencies between the two systems, the threshold for the determination of the homozygosity was set at ≥ 0.58, which is relatively low. However, this threshold accurately classified all the evaluated variants.

Among the variants identified in the study, the intronic mutation, NC_000012.12 (NM_000277.3):c.1066-11G > A was the most prevalent, detected in 15 out of 40 patients (37.5%). The second most frequently identified variant was NC_000012.12 (NM_000277.3):c.782G > A, found in 6 out of 40 patients (15%). Similar studies in the PKU patients from Türkiye underlined high frequencies of those variants, albeit with varying ratios [39–41]. Compound heterozygosity was more common than homozygosity, indicating that many patients carried different variants on each allele of the *PAH* gene. In three patients, a second variant necessary to establish compound heterozygosity could not be detected. This scenario suggests the presence of structural or deep intronic variants that were not covered by the sequencing systems used in this study. Finally, the incidence of PKU in Türkiye is relatively high, estimated at 1 in 6667 births [42]. This elevated incidence may be attributed to the high rate of consanguineous marriages among the parents of the patients, observed in 17 out of 40 patients (47.2%) in the present study. Consanguinity increases the likelihood of inherited recessive disorders like PKU manifesting in the offspring due to the increased chance of both parents carrying the same pathogenic variants.

## 5. Conclusion

We have developed and validated a novel in-house designed ONT-based assay for PKU diagnosis, achieving 100% concordance with Illumina-based results despite ONT's lower read quality. With comprehensive *PAH* gene coverage, long-read capabilities, and cost-effectiveness, the assay is well-suited for high-prevalence regions like Türkiye, where consanguinity increases PKU risk. The assay's rapid and reliable results support timely intervention, potentially improving neonatal screening and patient outcomes, especially in resource-limited settings. However, further cohort studies are needed to fully assess its efficacy in detecting structural and deep intronic variants due to the lack of such cases in the present study.

## Figures and Tables

**Table 1:** Demographic profile and *PAH* gene variants detected via ONT sequencing.

**Patient**	**Phe levels before treatment (*μ*Mol/L)**	**Clinical diagnosis (type of PKU)**	**Gender**	**Year of birth**	**Consanguinity**	**ONT variant**	**Heterozygosity (depth/allele frequency)**	**ACMG criteria**	**APV**	**GPV**	**Type of PKU according to BioPKU** ^**1**^
1	1074	mPKU	F	1997	No	NC_000012.12(NM_000277.3):c.782G > A	85/0.56	PM1, PM2, PM3, PM5, PP2, PP3, PS3	N/A	N/A	N/A
2	2400	cPKU	F	2002	No	NC_000012.12(NM_000277.3):c.781C > T	316/0.40	PVS1, PM2, PM3, PS3	0/0	0	Classic (12/12)
						NC_000012.12(NM_000277.3):c.473G > A	1729/0.45	PM1, PM2, PM3, PM5, PP2, PP3, PS3			
3	1440	cPKU	F	2002	No	NC_000012.12(NM_000277.3):c.781C > T	93/0.38	PVS1, PM2, PM3, PS3	0/0	0	Classic (12/12)
						NC_000012.12(NM_000277.3):c.473G > A	252/0.42	PM1, PM2, PM3, PM5, PP2, PP3, PS3			
4	1560	cPKU	M	1998	No	NC_000012.12(NM_000277.3):c.1066-11G > A	1400/0.62	PM2, PM3, PS3	0/0	0	Classic (673/681)
5	1464	cPKU	M	1998	No	NC_000012.12(NM_000277.3):c.1066-11G > A	594/0.60	PM2, PM3, PS3	0/0	0	Classic (673/681)
6	2058	cPKU	F	2002	No	NC_000012.12(NM_000277.3):c.1066-11G > A	897/0.62	PM2, PM3, PS3	0/0	0	Classic (673/681)
7	684	mPKU	M	1998	No	NC_000012.12(NM_000277.3):c.464G > A	1713/0.50	PM1, PM2, PM3, PM5, PP1, PP2, PP3, PS3	10/9,7	9.7	Mild HPA (3/3)
						NC_000012.12(NM_000277.3):c.898G > T	411/0.67	PM1, PM2, PM3, PM5, PP2, PP3, PS3			
8	1776	cPKU	M	1993	3^rd^-degree cousin	NC_000012.12(NM_000277.3):c.781C > T	257/0.38	PVS1, PM2, PM3, PS3	N/A	N/A	Classic (5/7)
						NC_000012.12(NM_000277.3):c.143 T > C	85/0.31	PM1, PM2, PM3, PP1, PP2, PP3, PS3			
9	1200	cPKU	M	1992	No	NC_000012.12(NM_000277.3):c.782G > A	146/0.32	PM1, PM2, PM3, PM5, PP2, PP3, PS3	1,5/0	1.5	Classic (93/123)
						NC_000012.12(NM_000277.3):c.1066-11G > A	786/0.33	PM2, PM3, PS3			
10	1050	cPKU	F	1996	No	NC_000012.12(NM_000277.3):c.782G > A	454/0.29	PM1, PM2, PM3, PM5, PP2, PP3, PS3	1,5/2	2	Classic (15/61)
						NC_000012.12(NM_000277.3):c.143 T > C	513/0.36	PM1, PM2, PM3, PP1, PP2, PP3, PS3			
11	>8 mg/dL by Guthrie test	cPKU	M	1993	Yes-unknown degree	NC_000012.12(NM_000277.3):c.754C > T	222/0.72	PM1, PM2, PM3, PM5, PP1, PP2, PP3, PS3	0/0	0	Classic (126/128)
12	1122	cPKU	M	2001	1^st^-degree cousin	NC_000012.12(NM_000277.3):c.782G > A	172/0.62	PM1, PM2, PM3, PM5, PP2, PP3, PS31	1,5/1,5	1.5	Classic (184/381)
13	1429	cPKU	F	1993	Yes-unknown degree	NC_000012.12(NM_000277.3):c.1066-11G > A	570/0.60	PM2, PM3, PS3	0/0	0	Classic (673/681)
14	1380	cPKU	M	1986	1^st^-degree cousin	NC_000012.12(NM_000277.3):c.842C > T	160/0.89	PM2, PM3, PM5, PP2, PP3, PS3	0/0	0	Classic (207/208)
15	2400	cPKU	M	2001	1^st^-degree cousin	NC_000012.12(NM_000277.3):c.1066-11G > A	570/0.76	PM2, PM3, PS3	0/0	0	Classic (673/681)
16	1500	cPKU	M	2001	1^st^-degree cousin	NC_000012.12(NM_000277.3):c.331C > T	1920/0.80	PVS1, PM2, PM3	0/0	0	Classic (28/29)
17	1800	cPKU	M	2002	1^st^-degree cousin	NC_000012.12(NM_000277.3):c.1159 T > C	321/0.87	PM1, PM2, PM3, PM5, PP2, PP3	0/0	0	Classic (2/2)
18	900	cPKU	F	Unknown	Unknown	NC_000012.12(NM_000277.3):c.592_613del	202/0.24	PVS1, PM2, PM3	N/A	N/A	N/A
						NC_000012.12(NM_000277.3):c.1169A > G	1383/0.40	PM1, PM2, PM3, PP2, PP3, PS3			
19	960	cPKU	M	1996	1^st^-degree cousin	NC_000012.12(NM_000277.3):c.842C > T	180/0.87	PM2, PM3, PM5, PP2, PP3, PS3	0/0	0	Classic (207/208)
20	1350	mPKU	M	1999	No	NC_000012.12(NM_000277.3):c.1169A > G	1120/0.69	PM1, PM2, PM3, PP2, PP3, PS3	6,8/6,8	6.8	Mild HPA (21/29)
21	1804	cPKU	F	2016	Unknown	NC_000012.12(NM_000277.3):c.168+5G > C	248/0.48	PM2, PM3, PP3	0/0	0	Classic (27/27)
						NC_000012.12(NM_000277.3):c.1066-11G > A	663/0.37	PM2, PM3, PS3			
22	1009	cPKU	M	2020	Unknown	NC_000012.12(NM_000277.3):c.168+5G > C	55/0.55	PM2, PM3, PP3	0/0	0	Classic (27/27)
						NC_000012.12(NM_000277.3):c.1066-11G > A	458/0.40	PM2, PM3, PS3			
23	1612	cPKU	F	2023	Unknown	NC_000012.12(NM_000277.3):c.1222C > T	1128/0.44	PM1, PM2, PM3, PM5, PP1, PP2, PP3, PS3	0/0	0	Classic (39/39)
						NC_000012.12(NM_000277.3):c.165del	367/0.51	PVS1, PM2, PM3			
24	960	mPKU	M	1999	No	NC_000012.12(NM_000277.3):c.533A > G	67/0.36	PM1, PM2, PM3, PM5, PP2, PP3, PS3	7,5/7,5	7.5	Mild HPA (1/3)
25	2460	cPKU	F	2001	3^rd^-degree cousin	NC_000012.12(NM_000277.3):c.1066-11G > A	860/0.76	PM2, PM3, PS3	0/0	0	Classic (673/681)
26	1380	cPKU	F	1995	No	NC_000012.12(NM_000277.3):c.47_48del	53/0.28	PVS1, PM2, PM3, PS3	N/A	N/A	N/A
						NC_000012.12(NM_000277.3):c.727C > T	212/0.38	PVS1, PM2, PM3, PS3			
27	870	mPKU	F	1999	3^rd^-degree cousin	NC_000012.12(NM_000277.3):c.143 T > C	109/0.34	PM1, PM2, PM3, PP1, PP2, PP3, PS3	2/0	2	Mild (30/49)
						NC_000012.12(NM_000277.3):c.1066-11G > A	1051/0.31	PM2, PM3, PS3			
28	3540	cPKU	F	1997	No	NC_000012.12(NM_000277.3):c.842C > T	245/0.89	PM2, PM3, PM5, PP2, PP3, PS3	0/0	0	Classic (207/208)
29	806	mPKU	M	2004	2^nd^-degree cousin	NC_000012.12(NM_000277.3):c.782G > A	259/0.58	PM1, PM2, PM3, PM5, PP2, PP3, PS3	1,5/1,5	1.5	Classic (184/381)
30	1266	cPKU	M	1997	No	NC_000012.12(NM_000277.3):c.1066-11G > A	2082/0.29	PM2, PM3, PS3	N/A	N/A	N/A
						NC_000012.12(NM_000277.3):c.169-13 T > G	3626/0.40	PM2, PM3⁣^∗^			
31	666	mPKU	F	1997	No	NC_000012.12(NM_000277.3):c.143 T > C	388/0.63	PM1, PM2, PM3, PP1, PP2, PP3, PS3	2/0	2	Classic (19/49)
32	2130	cPKU	F	1998	No	NC_000012.12(NM_000277.3):c.1066-11G > A	359/0.43	PM2, PM3, PS3	0/1,5	1.5	Classic (93/123)
						NC_000012.12(NM_000277.3):c.782G > A	122/0.18	PM1, PM2, PM3, PM5, PP2, PP3, PS3			
33	>8 mg/dL by Guthrie test	cPKU	M	1997	No	NC_000012.12(NM_000277.3):c.1066-11G > A	1158/0.31	PM2, PM3, PS3	0/0	0	Classic (673/681)
34	1800	cPKU	M	2000	1^st^-degree cousin	NC_000012.12(NM_000277.3):c.353-1G > A	383/0.81	PVS1, PM2, PP5	0/0	0	Classic (2/2)
35	2310	cPKU	M	1998	1^st^-degree cousin	NC_000012.12(NM_000277.3):c.754C > T	144/0.69	PM1, PM2, PM3, PM5, PP1, PP2, PP3, PS3	0/0	0	Classic (126/128)
36	ND	mPKU	F	1997	Yes-unknown degree	NC_000012.12(NM_000277.3):c.1169A > G	413/0.27	PM1, PM2, PM3, PP2, PP3, PS3	6,8/0	6.8	Mild HPA (13/13)
						NC_000012.12(NM_000277.3):c.1066-11G > A	442/0.28	PM2, PM3, PS3			
37	1362	mPKU	M	2001	2^nd^-degree cousin	NC_000012.12(NM_000277.3):c.898G > T	439/0.67	PM1, PM2, PM3, PM5, PP2, PP3, PS3	9,7/9,7	9.7	Mild HPA (25/25)
38	1800	cPKU	F	2000	1^st^-degree cousin	NC_000012.12(NM_000277.3):c.1087_1088del	1920/0.81	PVS1, PM2⁣^∗^	0/0	0	Classic (60/61)
39	2460	cPKU	M	2001	No	NC_000012.12(NM_000277.3):c.1068C > G	656/0.40	PVS1, PM2, PM3, PS3	N/A	N/A	N/A
						NC_000012.12(NM_000277.3):c.168G > T	108/0.46	PM2, PM3, PP2, PP3			
40	>8 mg/dL by Guthrie test	cPKU	F	1989	No	NC_000012.12(NM_000277.3):c.1066-11G > A	921/0.33	PM2, PM3, PS3	N/A	N/A	N/A
						NC_000012.12(NM_000277.3):c.724C > T	168/0.35	PM1, PM2, PM3, PP2, PP3			

*Abbreviations:* mPKU, mild phenylketonuria; cPKU, classic phenylketonuria; F, female; M, male; Phe, phenylalanine; N/A, not available; ONT, Oxford Nanopore Technology; APV, allellic phenotype value; GPV, genotypic phenotype value = APV (max) (0–2.7 is classic PKU, 2.8–6.6 is mild PKU and 6.7–10.0 is mild HPA).

⁣^∗^The variants are likely pathogenic according to the ACMG criteria.

^1^The most frequent type in the BioPKU database (http://www.biopku.org).

## Data Availability

All data generated or analyzed during the study are included in this manuscript. Further enquiries can be directed to the corresponding author.

## Supporting Information

Supporting Information Additional supporting information can be found online in the Supporting Information section. Figure S1. Representative agarose gel electrophoresis image showing PCR amplification results using primer pools. Lane 1: PCR with Pool 1 and reference human DNA. Lane 2: PCR with Pool 2 and reference human DNA. Lane 3: PCR with Pool 1 and negative control. Lane 4: PCR with Pool 2 and negative control. The molecular weight marker corresponds to 5 kb.
