# Supplementary figures and images for: An Oxford Nanopore Technologies–Based Sequencing Assay for Molecular Diagnosis of Phenylketonuria and Variant Frequencies in a Turkish Cohort
Source: Int J Genomics. 2025 Apr 25;2025:5552662. doi: 10.1155/ijog/5552662 (PMC12048195; doi:10.1155/ijog/5552662)

## Slide 1
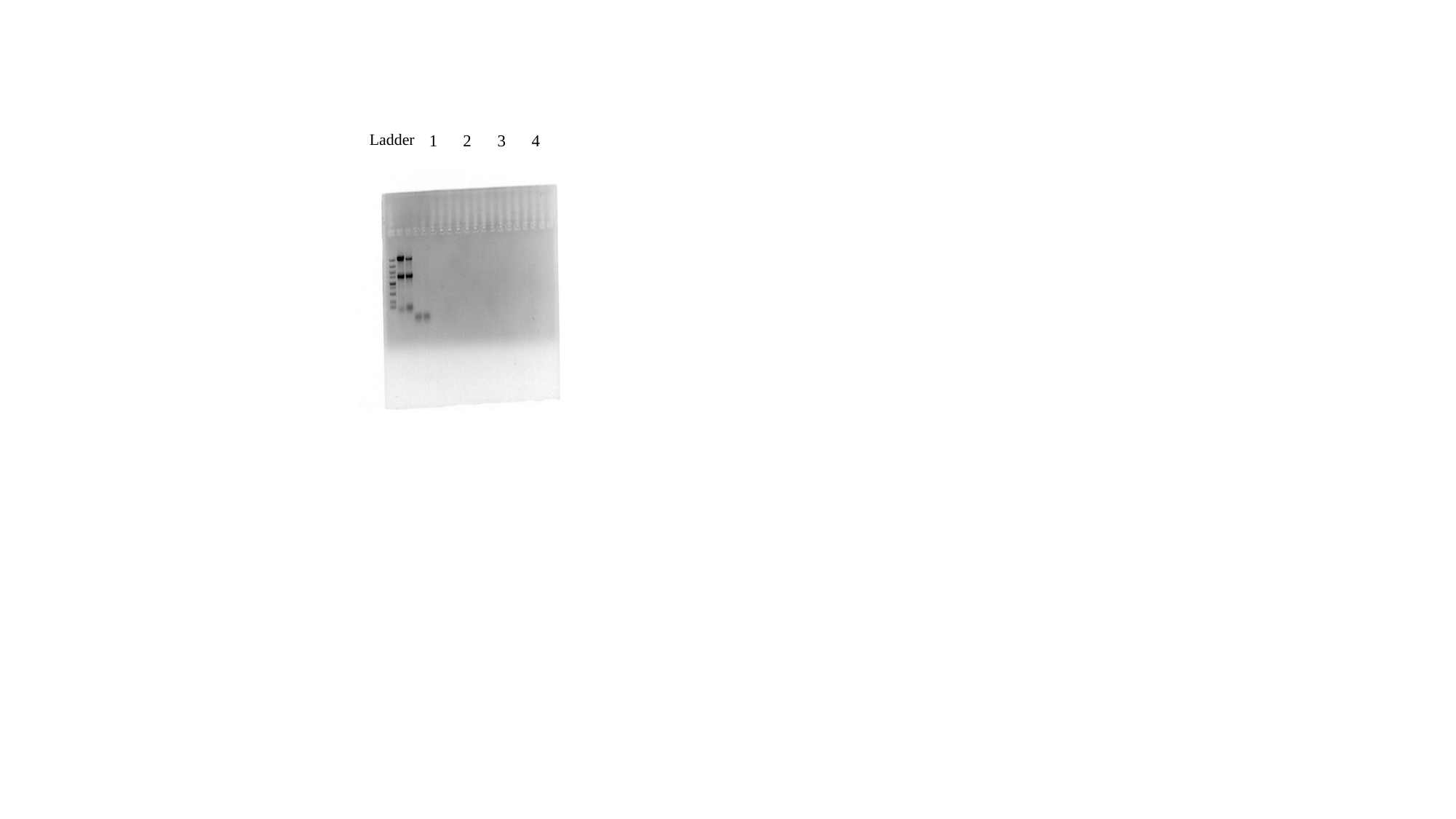

Ladder
1
2
3
4

Supplement: Supporting Information — Additional supporting information can be found online in the Supporting Information section. Figure S1. Representative agarose gel electrophoresis image showing PCR amplification results using primer pools. Lane 1: PCR with Pool 1 and reference human DNA. Lane 2: PCR with Pool 2 and reference human DNA. Lane 3: PCR with Pool 1 and negative control. Lane 4: PCR with Pool 2 and negative control. The molecular weight marker corresponds to 5 kb. [file 5552662.f1.pptx]
